# Review of "Cancer Nanotechnology: Methods and Protocols (Methods in Molecular Biology)" by **Stephen R. Grobmyer **(Editor), **Brij M. Moudgil **(Editor)

**DOI:** 10.1186/1475-925X-9-55

**Published:** 2010-09-24

**Authors:** Nicole F Steinmetz

**Affiliations:** 1 Department of Biomedical Engineering, Case Western Reserve University, Case Center for Imaging Research, 11100 Euclid Ave., Cleveland, OH, 44106, USA

## Abstract

Cancer remains one of the leading causes of death. Research and resulting technologies have contributed to rising numbers of cancer survivors. Cancer nanotechnology is a novel and burgeoning field with the promise to open the door for the development of improved cancer therapies and detection methods. Cancer nanotechnology has the potential to become clinical reality.

## Book Review

Cancer remains one of the leading causes of death. In 2009 562,340 fatalities were reported in the US. Cancer risk factors include genetics, environmental exposures (chemicals, infectious agents, radiation) as well as behaviors, and food choices. Research and resulting technologies have contributed to rising numbers of cancer survivors. In 2009, 11.4 million cancer survivors have been reported in the US; 1.6 million of these have survived cancer for more than 20 years (American Association for Cancer Research). High-throughput screening methods and databases contribute to a better understanding of the disease, earlier detection, and improved therapies.

Still, current detection methods do not reach the sensitivity required and chemotherapies are generally not targeted to the site of disease, resulting in undesired side effects. The goal of researchers is to develop targeted imaging modalities with higher signal-to-noise ratios and targeted devices for localized drug-delivery for improved efficacy and reduced adverse effects. Advances in nanotechnology, the exploitation of matter on the nanometer size-scale, now open the door to realize the development of such smart targeted devices. Nanomaterials have favorable properties for cancer therapy: i) the particles can carry a high "payload" of drugs or a "cocktail" of several drugs and or/imaging moieties, ii) nanoparticles can be engineered with targeting ligands that direct the materials and "payload" to the site of disease, and iii) the particle itself can have therapeutic or diagnostic properties.

The textbook "Cancer Nanotechnology" edited by Stephen R. Grobmeyer and Brji M. Moudgil (University of Florida) provides an interesting and valuable collection of cancer nanotechnologies. The focus lies in detailed descriptions of "how to" generate nanoparticle formulations for imaging and therapeutic applications. Various synthetic nanoparticle systems, their synthesis, characterization, as well as methods for *in vitro *and *in vivo *evaluation are described. It should be noted, that besides synthetic nanoparticles such as liposomes, metallic nanoparticles, and polymeric systems, biological scaffolds including nanobodies and viral nanoparticles have received growing attention over the past years.

"Targeting" is key in cancer nanotechnology. One differentiates between active vs. passive targeting. Passive targeting is based on hypervascularization of tumors; nanomaterials of 10-400 nm in size extravasate tumor vasculature through the enhanced permeability and retention (EPR) effect and thus accumulate in the tumor tissue. Methods to establish mouse tumor models and demonstration of EPR effects *in vivo *are described in chapter 3. Active targeting can be achieved on two levels: first, cell surface receptors that differentiate malignant cells from healthy cells can be targeted; this allows one to direct the nanocarrier to the site of disease (discussed in chapter 13); several ligand-receptor systems such as the folate receptor, aptamer systems, LHRH receptor, as well as magnetic targeting strategies are highlighted (chapters 16, 17, 18, 19). Second, targeting can be achieved on the molecular level inside the cell, here drugs are directed to interfere with biochemical pathways within the cells to either destroy the cancer cell or restore the cell's "software" to a normal state (chapter 2).

Nanomaterials hold great promise as imaging contrast agents to improve signal-to-noise ratios. Nanotechnology allows integration of different imaging methods through the use of multimodal agents. Throughout the book, medical imaging technologies are described including magnetic resonance imaging (MRI) and photoacoustic tomography (PAT). The synthesis and characterization of the following systems is discussed: Gd-chelated silica nanoparticles (used in MRI-PAT), gold cages and gold nanorods (used in PAT), and superparamagnetic iron oxide nanoparticles (used in PAT) (chapters 5, 6, 8, 19, 20, 23). Cancer therapies currently under development include i) targeted chemotherapy, ii) photothermal therapies (PTT), iii) hyperthermia therapy, and iv) photodynamic therapies (the latter is not described in "Cancer Nanotechnology"). The authors list a variety of materials: dielectric core-metal shell nanoparticles used for PTT, gold cages and nanorods used for PTT and hyperthermia, as well as liposomal doxorubicin and paclitaxel formulations that are used for improved chemotherapies (chapters 6, 7, 14, 23, 24). Each of these chapters describe the synthesis and characterization of the materials as well as their *in vitro *and *in vivo *testing.

Some nanomaterials are functional by themselves, e.g. gold nanoparticles can be used for PAT and PTT without further modification, other nanomaterials serve as carriers to direct imaging or therapeutic compounds toward the sites of disease. In these cases the carrier is loaded with imaging or therapeutic molecules and engineered with targeting ligands to direct the formulation to the tumor (alternatively passive targeting through EPR can be exploited). Chemical engineering of liposomes to act as drug-carriers, polymeric nanoparticular systems (including PLGA), silica nanoparticles for imaging, targeted gold immunoconjugates, drug-loaded dendrimers are introduced; protocols are described in extensive detail in chapters 9, 10, 11, 12 15, 20.

Overall, the book "Cancer Nanotechnology" describes state-of-the-art concepts to design and evaluate nanomaterials in vitro and in vivo using preclinical mouse models or clinical trials (chapter 25 & 26). The field is rapidly developing; a broad collection of materials is available. Liposomes and iron oxide nanoparticles have been FDA approved, many other materials are in (pre)clinical testing. Nanotechnology has the potential to revolutionize cancer detection and treatment. New technologies can lead to new breakthroughs, however, new technologies bring new risks. Nanotoxicology is an important topic. A database with reference protocols for the characterization of nanomaterials is currently under construction (a combined effort by the National Cancer Institute, National Institute of Standards and Technologies, and the Nanotechnology Characterization Laboratory) but not available yet. To fill this gap, the authors and editors put together a truly comprehensive and helpful reference guide listing methods for reporting particle size, porosity, materials composition, and surface properties (chapter 4).

The field of cancer nanotechnology has grown out of its infancy. Besides developing nanomaterials for *potential *applications, it is required to test and validate the designs and gain understanding on biodistribution and potential side effects; this can only be achieved using *in vivo *models. Therefore it is refreshing to see that an effort was made toward listing and references *in vivo *methods in addition to synthetic methods. Cancer nanotechnology is an exciting field; increasing knowledge is expected to open the door for the development of improved therapies and detection methods. Cancer nanotechnology has the potential to become clinical reality.

## Competing interests

The author declares that they have no competing interests.

